# High bone mass in mice can be linked to lower osteoclast formation, resorptive capacity, and restricted in vitro sensitivity to inhibition by stable sulforaphane

**DOI:** 10.1002/cbf.3734

**Published:** 2022-08-04

**Authors:** Polymnia Louka, Isabel R. Orriss, Andrew A. Pitsillides

**Affiliations:** ^1^ Skeletal Biology Group, Comparative Biomedical Sciences The Royal Veterinary College London UK

**Keywords:** bone mass, osteoclastogenesis, osteoclasts, SFX‐01, STR/Ort mice, sulforaphane

## Abstract

Mouse strains can have divergent basal bone mass, yet this phenotype is seldom reflected in the design of studies seeking to identify new modulators of bone resorption by osteoclasts. Sulforaphane exerts inhibitory effects on in vitro osteoclastogenesis in cells from C57BL/6 mice. Here, we explore whether a divergent basal bone mass in different mouse strains is linked both to in vitro osteoclastogenic potential and to SFX‐01 sensitivity. Accordingly, osteoclasts isolated from the bone marrow (BM) of C57BL/6, STR/Ort and CBA mice with low, high, and intermediate bone mass, respectively, were cultured under conditions to promote osteoclast differentiation and resorption; they were also treated with chemically stabilised sulforaphane (SFX‐01) and respective sensitivity to inhibition evaluated by counting osteoclast number/resorption activity on dentine discs. We observed that osteoclastogenesis exhibited different macrophage colony‐stimulating factor/receptor activator of nuclear factor kappa‐Β ligand sensitivity in these mouse strains, with cells from C57BL/6 and CBA generating higher osteoclast numbers than STR/Ort; the latter formed only half as many mature osteoclasts. We found that 100 nM SFX‐01 exerted a potent and significant reduction in osteoclast number and resorptive activity in cells derived from C57BL/6 mice. In contrast, 10‐fold higher SFX‐01 concentrations were required for similar inhibition in CBA‐derived cells and, strikingly, a further 2.5‐fold greater concentration was required for significant restriction of osteoclast formation/function in STR/Ort. These data are consistent with the notion that the BM osteoclast precursor population contributes to the relative differences in mouse bone mass and that mice with higher bone mass exhibit lower in vitro osteoclastogenic potential as well as reduced sensitivity to inhibition by SFX‐01.

## Significance statement

This work investigated whether osteoclast formation and activity differ in bone marrow cells derived from mouse strains with high bone mass, and also examined their sensitivity to SFX‐01. The key findings were that in vitro osteoclastogenesis and resorption, as well as sensitivity to macrophage colony‐stimulating factor (M‐CSF)/receptor activator of nuclear factor kappa‐Β ligand (RANKL) were lower in cells derived from mice with high bone mass. Furthermore, that SFX‐01 exerts lower antiosteoclastogenic/resorptive effects in these cells. This links in vitro osteoclast multi nucleation behaviour to in vivo bone mass. The distinct sensitivity to both the pro‐osteoclastogenic cytokines, M‐CSF and RANKL, and the antiosteoclastogenic activity of SFX‐01, makes it tempting to propose that SFX‐01 has potential as a therapeutic candidate in bone diseases such as osteoporosis.

## 1 INTRODUCTION

Bone mass is established and maintained by a balancing of the bone formation and resorption phases.^1^ Modification to this steady state, involving greater (or lesser) levels of osteoclast formation or resorptive activity will result in low (or high) bone mass phenotypes. Thus, resorption by osteoclasts during (re)modelling exerts a vital role in the many skeletal diseases associated with modified bone mass.^2^ Likewise, it is known that bone formation by osteoblasts can also be linked to shifts in this balance, with human high bone mass syndromes, for example, arising via modifications in Wnt or TGF‐β signalling.^3^ The extent to which bone mass, which is the product of the balanced action of resorption and formation in vivo, can be linked to the in vitro behaviour of either the constituent osteoclasts or osteoblasts is incompletely defined.

A conserved feature of the osteoclast formation and maturation stages both in vivo and in vitro is the vital stimulatory roles served by macrophage colony‐stimulating factor (M‐CSF) and receptor activator of nuclear factor kappa‐Β ligand (RANKL). M‐CSF mainly promotes the proliferation and survival of osteoclast precursors, and RANKL primarily drives their osteoclast differentiation.^4^ In vitro osteoclast cultures, using mouse bone marrow (BM) as a source of precursors seeded on dentine, provide an excellent resource for studying the formation, multinucleation, differentiation, and resorption stages. Culturing precursors on plastic, in contrast, gives a limited capacity to sequentially resolve these phases.^5^ While there is a wealth of in vivo evidence linking osteoclast function to bone mass regulation in humans and mice, the possibility that bone mass in mice, which serve as the source of osteoclasts, will be linked to conserved differences in osteoclastogenic and functional in vitro behaviours has only been explored once before.^6^

Mouse strains have been critical in the experimental exploration of bone mass regulation and in defining the factors vital in controlling bone cell function. Most studies use inbred mice, as they effectively provide identical phenotypically normal ‘twins’ in which insights into the genetic basis of variation in bone density can be gained. When compared to 10 other inbred strains, including C3H/HeJ, DBA/2J, and BALB/c, the widely used C57BL/6 strain was found to possess relatively low cortical bone density.^7^ In contrast, the inbred STR/Ort mouse that develops spontaneous osteoarthritis (OA) from ∼20 weeks of age^8^, ^9^ is known to exhibit a very high bone mass phenotype.^10^ Cortical and trabecular bone mass in STR/Ort mice is indeed higher than in the parental CBA strain, even though their adult weight does not differ significantly, and despite the latter exhibiting greater bone mass relative to many other inbred strains for example, C57BL/6.^11^, ^12^, ^13^ Indeed, bone mass in STR/Ort is high enough to generate BM space compression and to lead to extramedullary hematopoiesis and changes in the behaviour of osteoclasts in vivo.^14^ Together, the C57BL/6, CBA, and STR/Ort strains provide for a range of in vivo bone mass phenotypes that allow for conserved links to differences in osteoclastogenic and functional in vitro behaviours to be examined.

To date, only one study has explored this link between in vivo mouse bone mass and the in vitro behaviour of osteoclast precursors. Linkhart et al.^6^ found that BM from mice, which had lower femoral bone density than C3H/HeJ, yielded more osteoclasts and resorbed dentine more extensively in vitro. While these studies were performed using coculture with Swiss/Webster mouse osteoblasts as the ‘osteoclastogenic drive’ (conducted before RANKL/M‐CSF were available commercially) they nonetheless support the hypothesis that precursors derived from mice with divergent bone mass may also exhibit differential sensitivity to M‐CSF‐/RANKL‐induced osteoclastogenesis and resorption capacity in vitro.

Appreciation of the role of osteoclasts in skeletal pathology has yielded pharmacological agents that modulate bone mass. Approved agents (e.g., bisphosphonates, selective oestrogen receptor modulators, and anti‐RANKL antibodies) while effective, have side effects^15^ and, hence, a considerable demand for alternative treatment approaches remains. Powerful antioxidants are an alternative to achieve beneficial bone effects and the avoidance of osteoporotic bone loss.^15^, ^16^ Sulforaphane (SFN) is a natural antioxidant found at high levels (as glucoraphanin) in cruciferous vegetables. SFN activates the NRF2 pathway and has anti‐inflammatory effects,^17^ protecting against oxidative stress in many cell types.^18^

SFN is inherently unstable, rendering it nonviable as a pharmaceutical agent; its chemical stabilisation by addition of a synthetic alpha‐cyclodextrin moiety has led to the generation of Sulforadex^TM^ (SFX‐01). SFN is known to suppress osteoclastogenesis^19^, ^20^, ^21^, ^22^ but one study has demonstrated that SFX‐01 can exert similar actions,^11^ and the extent to which this may depend on the murine source of osteoclast progenitors is not defined. Here, we explore whether BM precursors derived from mice with divergent bone mass (C57BL/6, CBA, STR/Ort) display differing sensitivity to M‐CSF/RANKL resulting in altered osteoclastogenic potential in vitro. Whether this sensitivity extends concentration‐dependently to inhibitory effects of SFX‐01 on osteoclast formation and function will also be investigated.

## 2 MATERIALS AND METHODS

### 2.1 Animals

All of the procedures conducted in the facility were in accordance with the Animals Act (Scientific Procedures) 1986 and approved by the Royal Veterinary College (RVC) Research Ethics Committee. Primary osteoclasts were isolated from C57BL/6, CBA, and STR/Ort strains. Mice were housed at 21 ± 2°C with 12‐h light/dark cycles with free access to food and water.

### 2.2 Osteoclast formation and resorption in vitro

**Figure 1:**
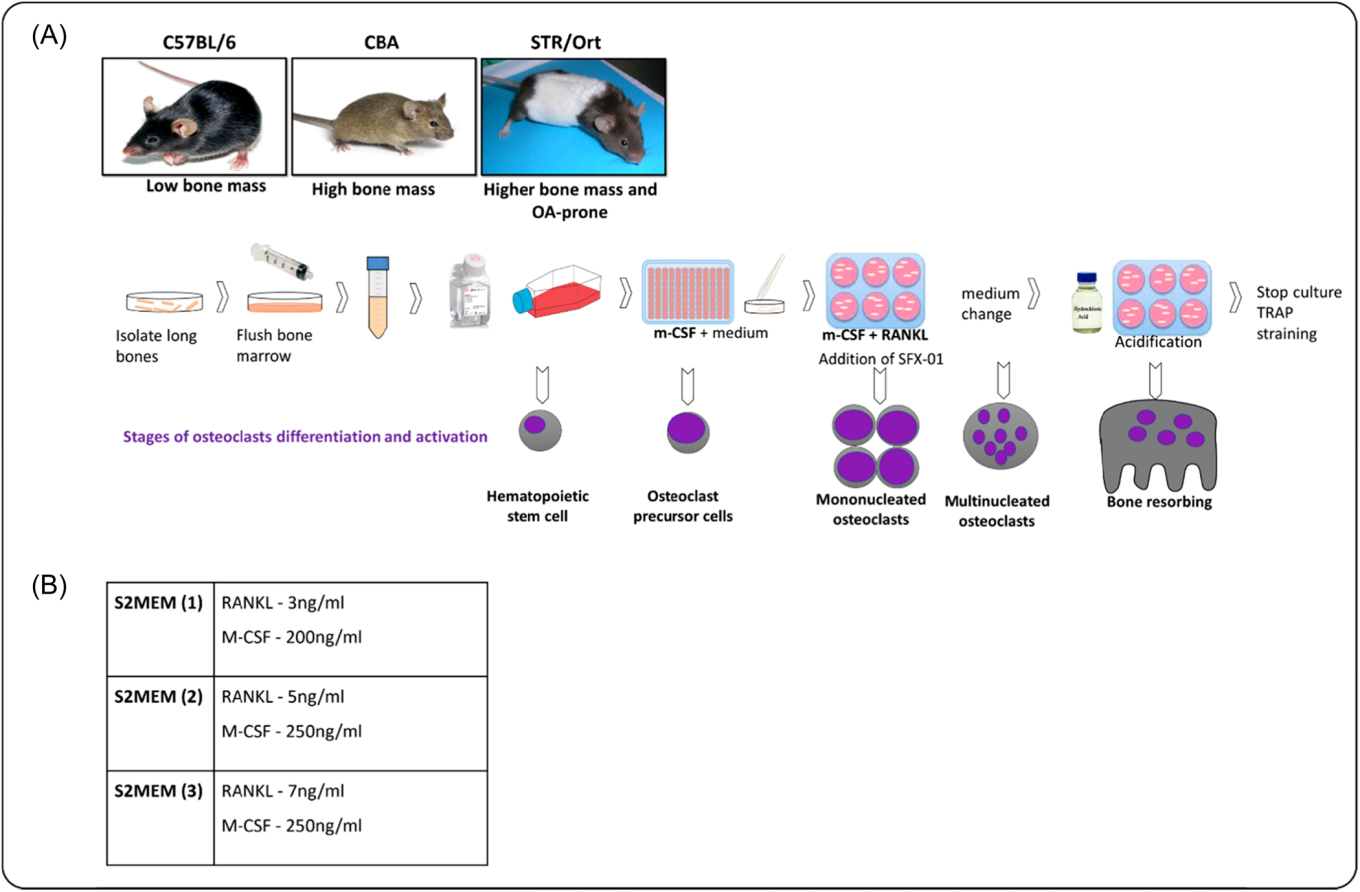
Schematic illustration of murine osteoclast differentiation and activation. (A) Bone marrow‐derived cells undergo differentiation into osteoclast precursors in the presence of M‐CSF which primarily promotes preosteoclast proliferation and survival. M‐CSF and RANKL, in combination, are required for differentiation of mononuclear precursors into multinucleated and mature osteoclasts. Osteoclast‐mediated bone resorption is initiated 5 days after the initial marrow isolation by acidifying the S2MEM medium in which the cells were maintained to pH 6.9. When included, SFX‐01 was added to the medium from Days 2 to 7. (B) Range of RANKL and M‐CSF concentrations used in supplementation of the S2MEM medium for the determination of optimal primary osteoclast formation from precursor cells derived from three different mice strains in culture. M‐CSF, macrophage colony‐stimulating factor; RANKL, receptor activator of nuclear factor kappa‐Β ligand.

The long bones were dissected from 6 to 10‐week‐old mice from each strain. Bones were cut across the epiphyses and the marrow was flushed out with phosphate buffered saline (PBS) as previously described.^5^, ^23^ Briefly, the resultant BM suspension was then centrifuged at 1500 rpm and resuspended in MEM supplemented with 10% foetal calf serum (FCS), 2 mM l‐glutamine, 100 U/ml penicillin, 100 μg/ml streptomycin and 0.25 μg/ml amphotericin, 100 nM prostaglandin E_2_ (PGE_2_) and 50 ng/ml M‐CSF (Figure 1A). The cell suspension was then cultured for 24 h in 75 cm² flasks 5% CO_2_/95% atmospheric air. The next day (Day 1), the nonadherent cell suspension was removed, centrifuged, and resuspended in MEM containing 10% FCS, 2 mM l‐glutamine, 100 U/ml penicillin, 100 μg/ml streptomycin and 0.25 μg/ml amphotericin,100 nM PGE_2_ and 200 ng/ml M‐CSF as well as 5 ng/ml RANKL (R&D Systems Europe Ltd.)—referred to as S2MEM, see below. Cells were plated onto 5 mm diameter dentine discs (10_6_ cells/disc) in 96‐well plates and incubated overnight at 37°C/5% CO_2_ to allow for attachment of osteoclast precursors. After 24 h (Day 2), the dentine discs were transferred to six‐well plates (3–4 discs/well in 3 ml S2MEM) and maintained for 3 days. S2MEM was acidified to pH 6.9 (Day 5) by addition of HCL to activate osteoclast resorption for 48 h (Day 7). In some experiments, SFX‐01 (in PBS; PharmAgra Labs) was added at the stated concentration (100 nM to 1 μΜ) for the duration of the culture; PBS served as the vehicle control (Figure 1A).

As outlined (above), standard M‐CSF and RANKL concentrations for S2 media are 200 and 3 ng/ml, respectively. However, to determine if these levels were sufficient to generate osteoclasts from all three mouse strains, these cytokines were also tested at a range of concentrations (Figure 1B). All other medium supplements remained the same. During this optimisation, the total number of nonadherent marrow cells isolated from the bones of each mouse strain (Day 1) was evaluated to establish relative cell yield.

### 2.3 Analysis of osteoclast formation and bone resorption

Discs containing adherent cells/osteoclasts were fixed in 2% glutaraldehyde and stained to demonstrate tartrate‐resistant acid phosphatase (TRAP) activity. Osteoclasts were defined as TRAP‐positive cells containing two or more nuclei and/or clear evidence of resorption pit formation. Osteoclast number and the area resorbed on each disc were assessed ‘blind’ by transmitted light microscopy and reflective light microscopy and dot‐counting morphometry using Image J, respectively.^5^

### 2.4 Statistics

All statistical analysis was performed using GraphPad Prism 8. To show the difference between two groups, a two‐tailed paired *t*‐test was performed. Between three or more groups, a repeated measures one‐way analysis of variance or a repeated measures mixed‐effects model was performed with Fisher's LSD post hoc analysis. Each individual reported set of data represents *n* = 4–5 biological ‘experimental’ replicates, in each of which there were 6–8 technical replicates (number of discs). Those biological experiments were then combined and presented as a single graph with biological replicates as mean ± SEM using cells derived from different animals.

## 3 RESULTS

### 3.1 Fewer BM cells can be derived from mice with higher bone mass

Initial studies focused on measuring the total number of nonadherent marrow cells that could be isolated from each mouse strain (on Day 1, see above; Figure 1). Using 8–10‐week‐old STR/Ort mice for extraction proved difficult due to the narrowness of the marrow space and led to minimal cell yields. C57BL/6 and CBA mice at similar ages (8–10‐week‐old) readily provided a source of marrow and yielded cell numbers sufficient for subsequent osteoclast cultures. For this reason, further studies used marrow‐derived from mice at ∼6 weeks of age.

**Figure 2:**
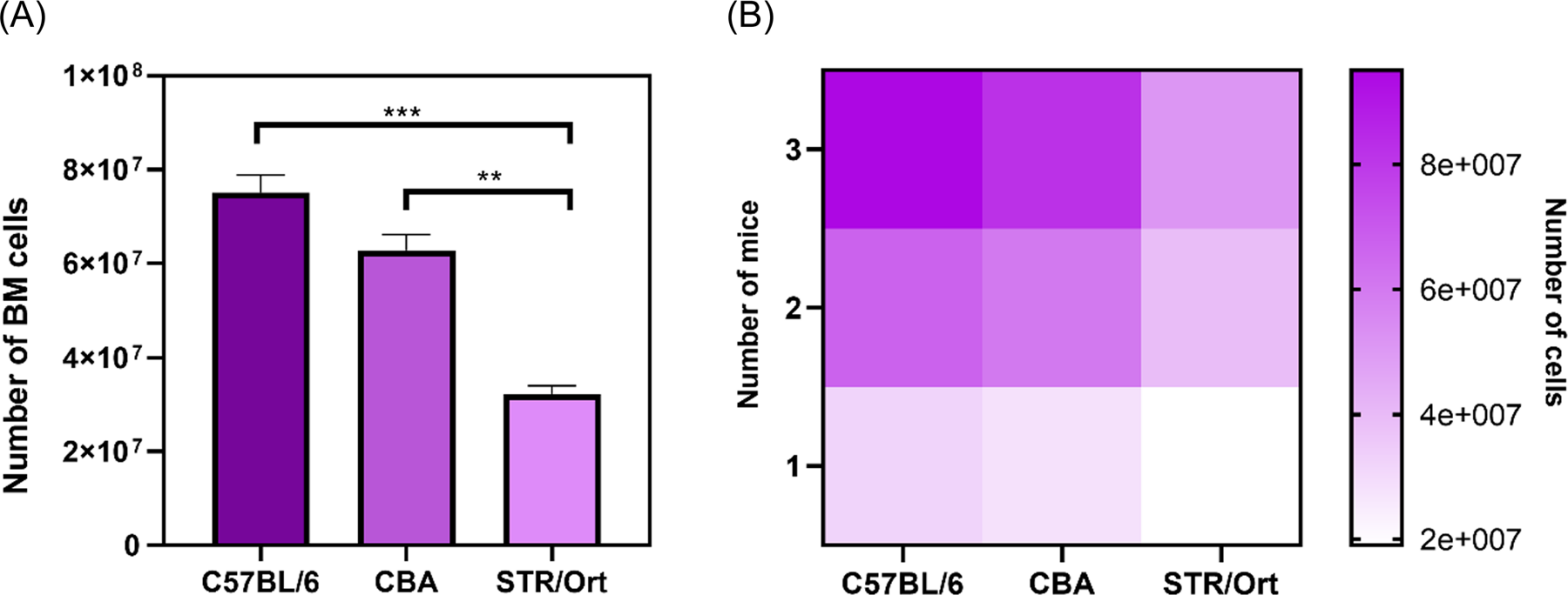
(A) Cell yield from C57BL/6, CBA and STR/Ort mouse BM differs. BM cell yield extracted from STR/Ort mice is significantly lower than from C57BL/6 (*p* < .001) and CBA mice (*p* < .01). **p ≤ .01 ***p ≤ .001. Combined data from *n* = 5 experiments, 6–8 replicates/condition. (B) The heatmap, represents the number of mice required for each mouse strain and the yield of BM cells that can be extracted in each case. As indicated, STR/Ort yield extraction requires double the number of mice, compared with C57BL/6 and CBA. BM, bone marrow.

Despite this switch to younger 6‐week‐old mice, collection from STR/Ort animals generated markedly lower cell yield than C57BL/6 or CBA mice; total BM cell number was ∼4‐fold and ∼2‐fold greater in C57BL/6 (*p* < .001) and CBA mice (*p* < .05), respectively (Figure 2). Evaluation of cell yield from a single mouse of each strain showed (Figure 2A) that the bones from a C57BL/6 mouse generated up to ${2\times 10}^{7}\;$cells, while at least two STR/Ort mice were required to achieve similar yields (Figure 2B). Further studies therefore consistently used double the number of STR/Ort mice for BM isolation compared to C57BL/6 and CBA mice.

### 3.2 Pre‐established conditions (M‐CSF and RANKL) are not sufficient for the generation of mature osteoclasts in all mouse strains

**Figure 3:**
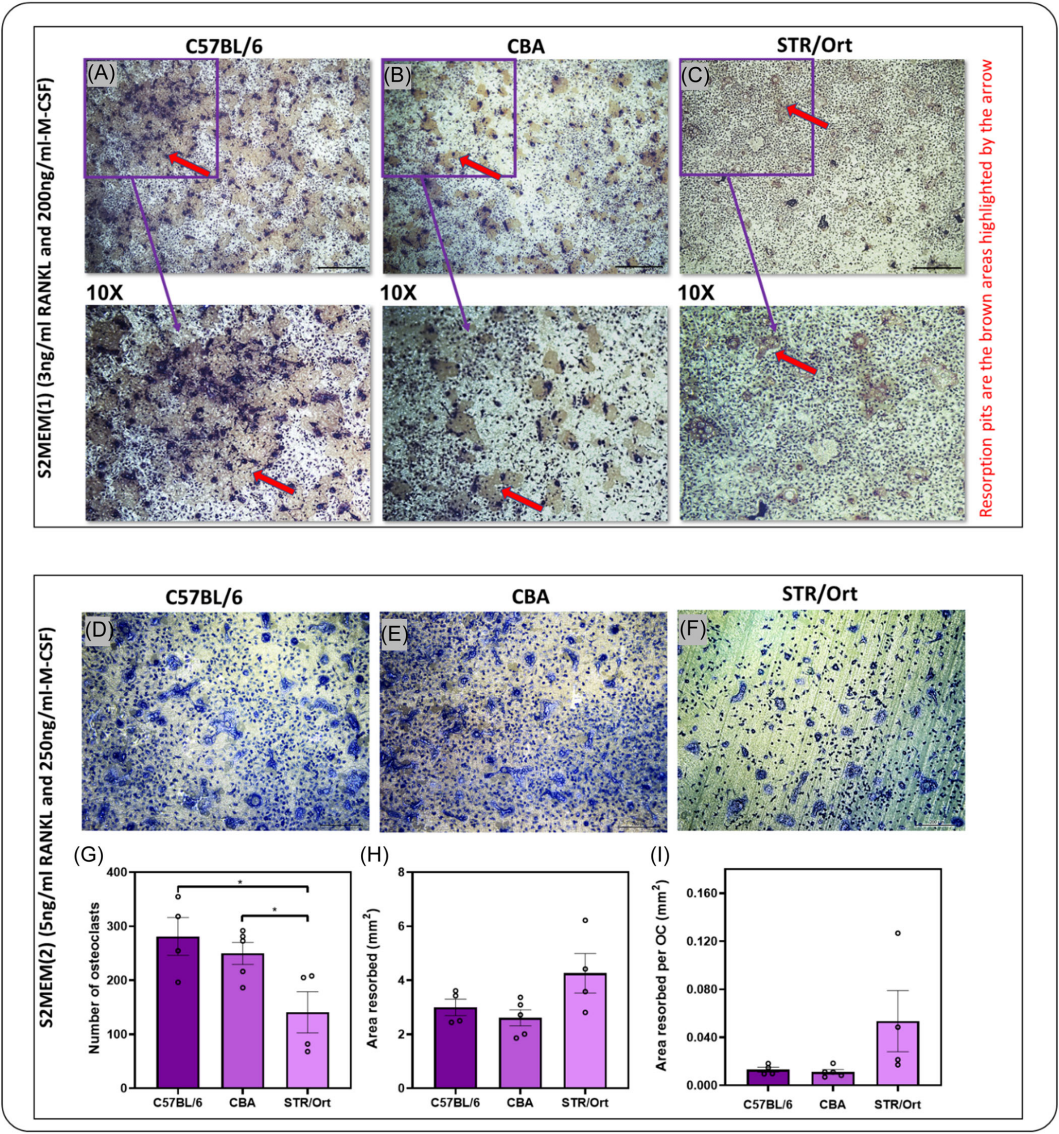
There is lower sensitivity to M‐CSF‐/RANKL‐induced in vitro capacity in BM sources from the STR/Ort mice with the highest bone mass. (A–C) Representative reflective light images of osteoclasts extracted from STR/Ort (highest bone mass), CBA (high bone mass), and C57BL/6 (low bone mass) indicate that STR/Ort mice failed to produce mature osteoclasts and resorb using the well‐established culture conditions of S2MEM (1) (3 ng/ml RANKL and 200 ng/ml M‐CSF). (D–F) Representative toluidine blue images indicating BM cells extracted from the high bone mass STR/Ort need raised cytokines (S2MEM (2) (5 ng/ml RANKL and 250 ng/ml M‐CSF) to differentiate and form mature osteoclasts. (G–I) Quantitative analysis indicates reduced osteoclast numbers (TRAP+) in BM‐derived from high bone mass STR/ort mice compared with C57BL/6 and CBA using the optimal conditions of S2MEM (2) (5 ng/ml RANKL and 250 ng/ml M‐CSF) and no effect in resorption or area resorbed/OC. Each experiment was repeated 4–5 times, with 6–8 replicates within each experiment. Numbers are also expressed as mean osteoclast per dentine disc ± SEM, *Statistical *p* ≤ .05. BM, bone marrow; M‐CSF, macrophage colony‐stimulating factor; RANKL, receptor activator of nuclear factor kappa‐Β ligand; TRAP, tartrate‐resistant acid phosphatase.

Differences in sensitivity to varying M‐CSF and RANKL concentrations were evaluated in cells from all three mouse strains plated at identical densities. Using well‐established conditions (3 ng/ml RANKL and 200 ng/ml M‐CSF), BM cells derived from C57BL/6 efficiently underwent osteoclast differentiation and multinucleation (Figure 3A). Although marrow cells from CBA mice also showed some osteoclast differentiation and multinucleation under these conditions, their behaviour exhibited some insufficiency in osteoclastogenic capacity even upon exposure to RANKL and M‐CSF (Figure 3B). In contrast, BM cells from STR/Ort mice failed to produce mature osteoclasts, with cultures containing significant mononuclear precursors but very few, if any, mature multinucleated osteoclasts (Figure 3C).

To confirm whether the relative lack of differentiation/multinucleation in precursors derived from CBA and STR/Ort mice was due to reduced sensitivity to M‐CSF and RANKL, BM cells from all three strains were additionally cultured in a medium containing raised cytokine concentrations (see Figure 1B; S2MEM (2) and S2MEM (3)). Results revealed that an S2MEM (2) medium containing 5 ng/ml RANKL and 250 ng/ml M‐CSF generated the greatest number of large, mature TRAP+ osteoclasts in cells derived from all three mouse strains (Figure 3D–F), while visual examination of S2MEM (3) failed to produce optimal levels for all three mice strain (data not shown). Thereby, using S2MEM (2), it was found that the number of osteoclasts on dentine was significantly greater (*p* < .05) in cells derived from C57BL/6 and CBA mice compared to STR/Ort mice; no differences between C57BL/6 and CBA were observed (Figure 3G). Interestingly, even though there was a trend for higher total resorption and resorption per osteoclast in cells derived from STR/Ort mice compared with C57BL/6 and CBA, this was not statistically significant, indicating that SFX‐01 exerted more profound effects in regulating osteoclastogenesis rather than resorption (Figure 3H–I).

### 3.3 SFX‐01 exerts lower antiosteoclastogenic effects in cells from mice with higher bone mass

**Figure 4:**
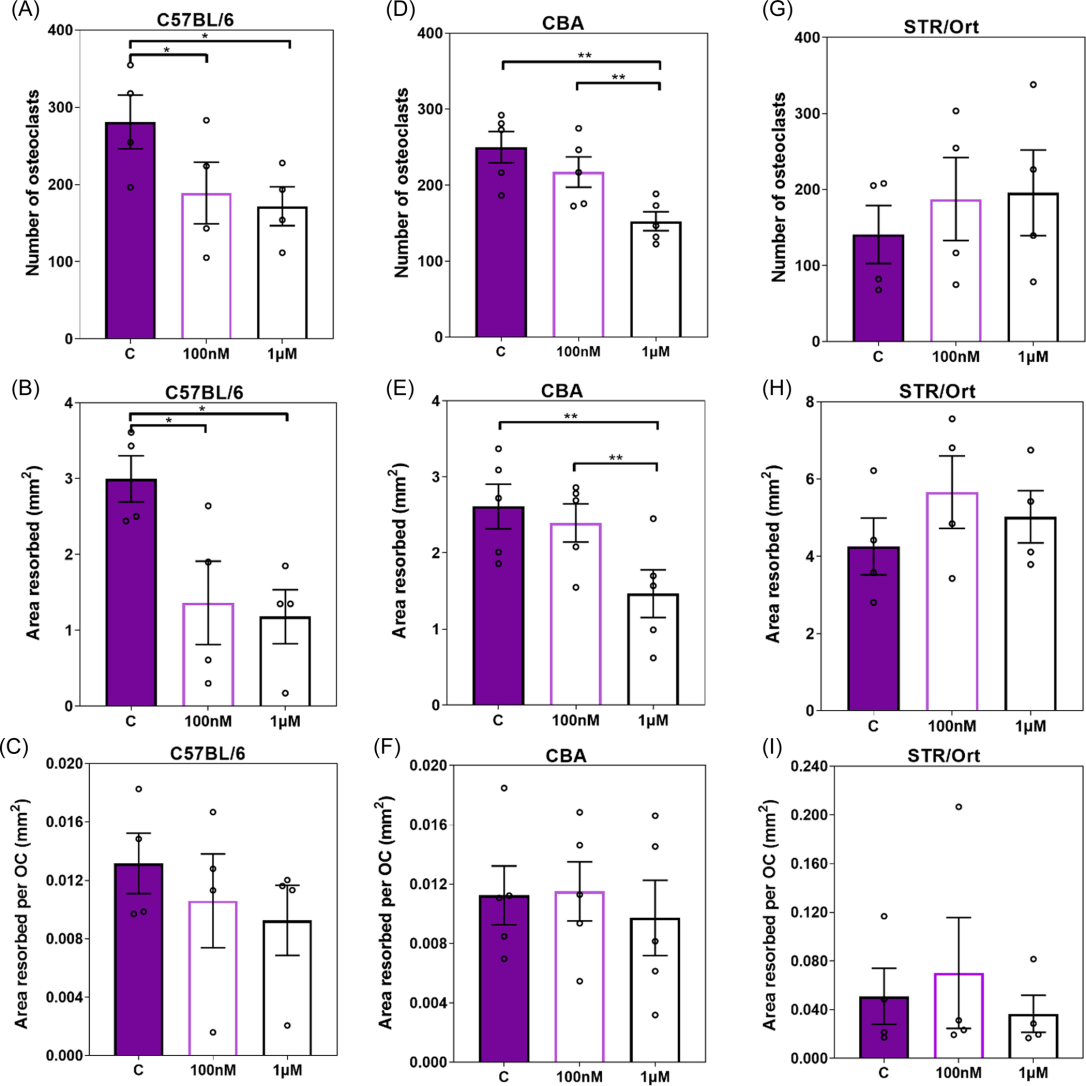
Effect of different SFX‐01 concentrations on TRAP+ osteoclast numbers, area resorbed, area resorbed/OC from C57BL/6, CBA, and STR/Ort mice. 100 nM was sufficient to inhibit osteoclastogenesis (A) and total area resorbed (B), but not area rersobed per/OC (C) in cells derived from C57BL/6; while for CBA 1 μΜ was required to achieve similar inhibition levels (D, E, F); STR/Ort derived osteoclasts were completely insensitive up to this concentration(G, H, I). *p ≤ .05, **p ≤ .01. Combined data from *n* = 5 experiments, 6–8 replicates/condition. TRAP, tartrate‐resistant acid phosphatase.

**Figure 5:**
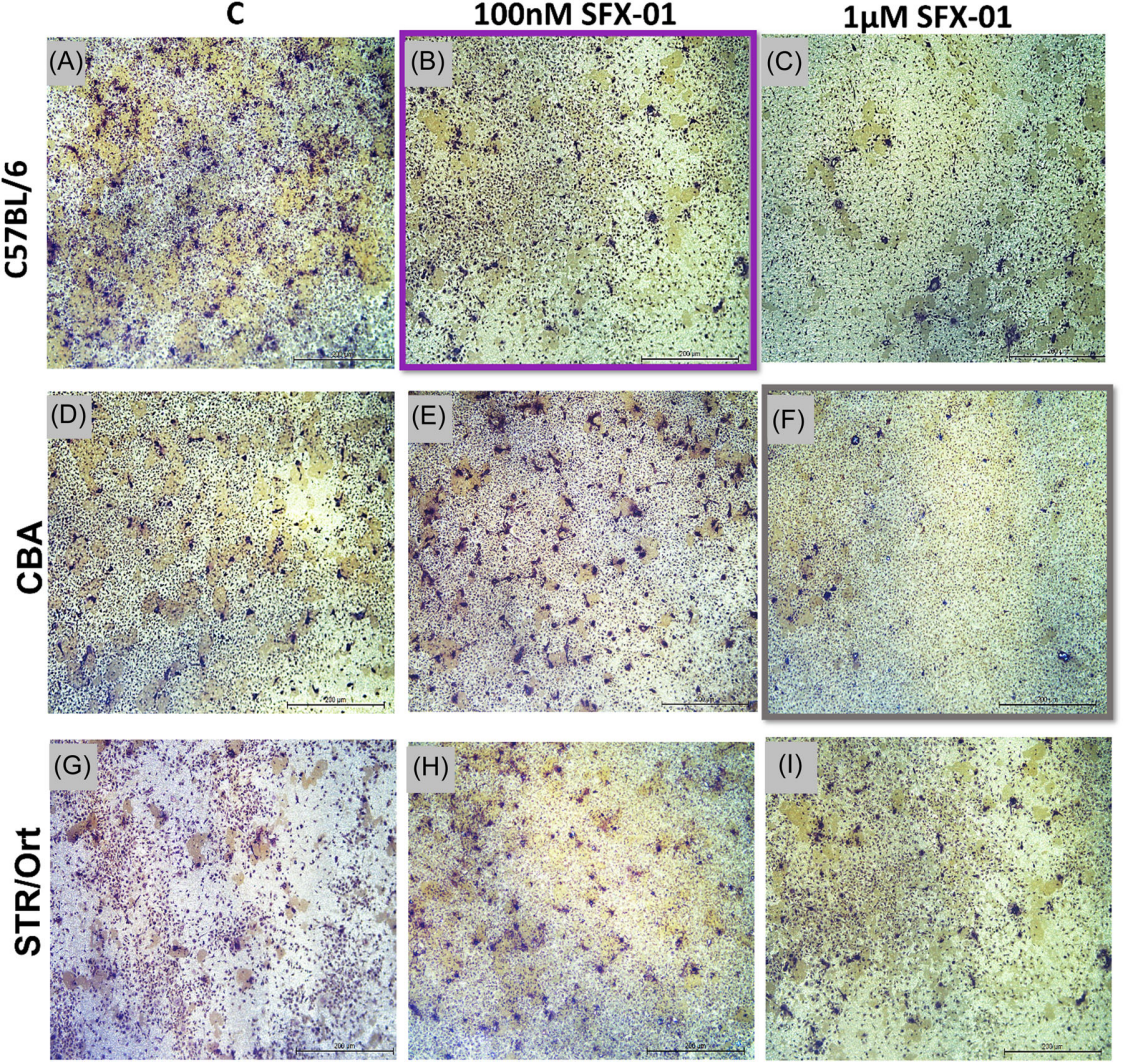
(A–I) Representative reflective light images of osteoclasts extracted from STR/Ort, CBA, and C57BL/6 mice treated with 100 nM to 1 μM SFX‐01. BM cells extracted from C57BL/6, CBA, and STR/Ort were treated with PBS (controls A, D, and G), 100nM SFX‐01 (B, E, and G, respectively), and 1 μΜ SFX‐01 (C, F, and I). Highlighted images (purple and grey boxes) show the concentration of SFX‐01 required to inhibit osteoclastogenesis. 100 nM of SFX‐01 was required for BM cells extracted from C57BL/6 and 1 μΜ for BM cells extracted from CBA. Scale bar = 200 μm. BM, bone marrow.

To determine whether this reduced osteoclastogenic potential in the high bone mass strains extends to a modulatory effect of SFX‐01 on osteoclast formation and function, the osteoclast number/resorptive capacity was assessed in response to a range of SFX‐01 concentrations (100 nM to 2.5 μM) in each mouse strain. In C57BL/6, SFX‐01 (≥100 nM) decreased osteoclast number and total area resorbed; area resorbed per osteoclast was unchanged (Figures 4A–C and 5B). Higher SFX‐01 concentrations (≥1 μΜ) were required to achieve similar levels of inhibition of osteoclastogenesis and resorption in CBA mice (Figures 4D–F and 5F). Osteoclasts derived from STR/Ort mice exhibited no significant modification in the number or resorptive capacity in response to SFX‐01 (≤1 μΜ) (Figures 4G–I and 5G–I). This is consistent with the notion that the sensitivity to SFX‐01‐mediated inhibition of osteoclast formation and function is related to in vivo bone mass in these mouse strains.

**Figure 6:**
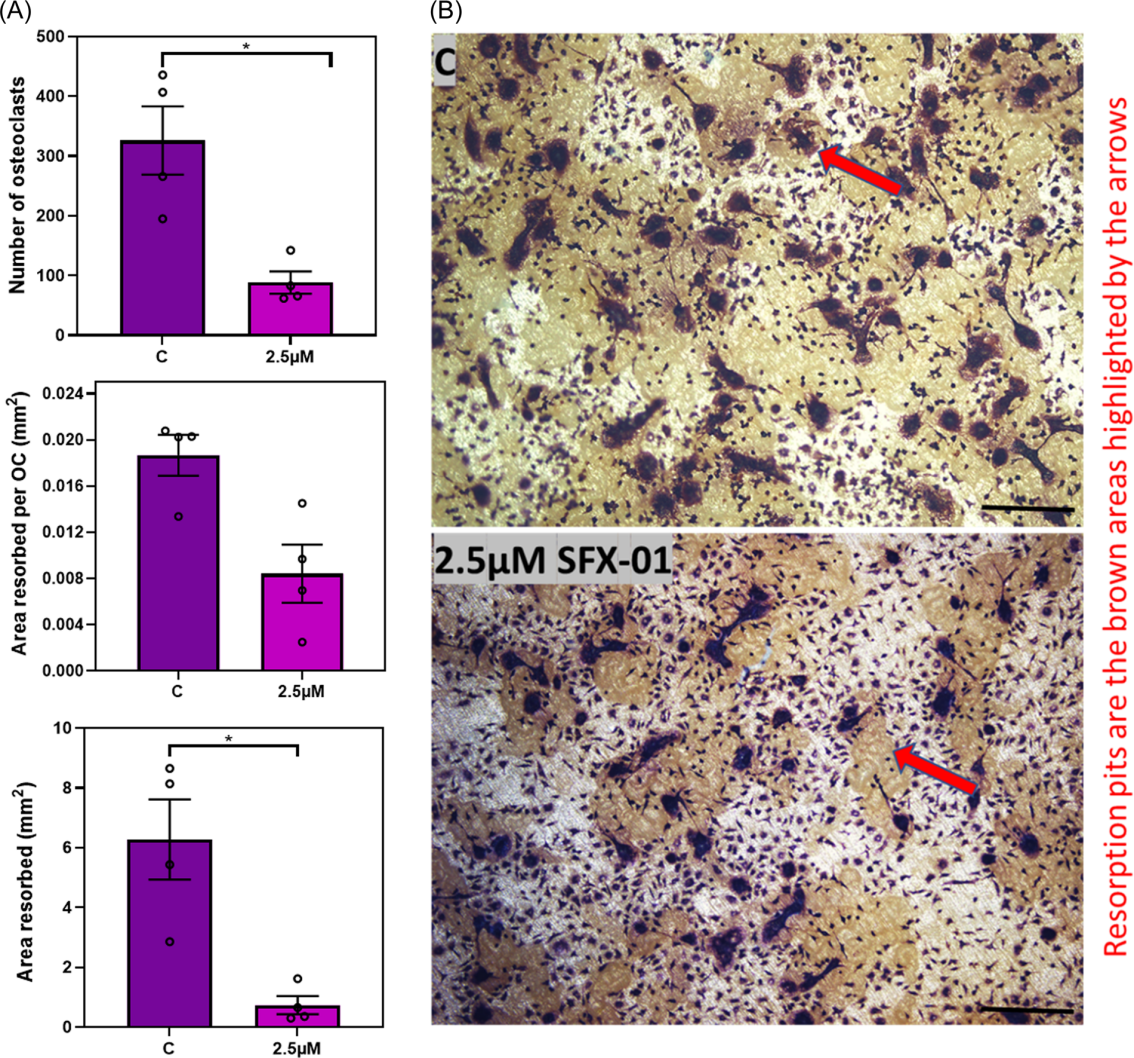
BM cells extracted from STR/Ort mice require higher concentration of SFX‐01 to inhibit osteoclastogenesis. (A) Effect of 2.5 μΜ SFX‐01 on TRAP+ osteoclast number, area resorbed and area resorbed/OC from STR/Ort mice. (B) Representative reflective light images of osteoclasts extracted from BM of STR/Ort mice; 2.5 μΜ of SFX‐01 was required for inhibition of osteoclastogenesis/resorption. Resoprtion pits are the brown areas highlighted by the red arrows Combined data from *n* = 4 experiments, six–eight replicates for each group. *p ≤ .05. BM, bone marrow; TRAP, tartrate‐resistant acid phosphatase.

Further studies using STR/ORT cells, found that 2.5 μΜ SFX‐01 was sufficient to reduce both osteoclast number and their total resorptive capacity (Figure 6). High 10 μΜ SFX‐01 concentrations almost abolished osteoclastogenesis in all mouse strains (data not shown). Together these data show that SFX‐01 exerts more significant suppression of the total area resorbed than the resorption achieved per osteoclast, which suggests that SFX‐01 preferentially targets earlier osteoclastogenic differentiation.

## 4 DISCUSSION

The in vitro findings show that: (i) initial yield of nonadherent marrow cells; (ii) their sensitivity to M‐CSF/RANKL driven osteoclast differentiation, (iii) the number of TRAP+ multinucleated osteoclasts under optimal conditions, as well as (iv) their sensitivity to exogenous SFX‐01 are all significantly, and progressively lower in mice with successively greater in vivo bone mass. These findings suggest that BM cells derived from animals with a high in vivo bone mass are less sensitive to M‐CSF and RANKL in vitro leading to lower osteoclastogenesis. They also support the hypothesis that similar sensitivity extends to the inhibitory effects of SFX‐01 on osteoclast formation/function and show, for the first time, that direct osteoclast‐targeted actions of this pharmacologically deliverable, stabilised form of SFN, extend across a range of inbred mouse strains. Finally, the lack of effect upon the area resorbed by each individual mature osteoclast suggests that the effects of SFX‐01 are more profound in controlling osteoclastogenesis.

In vitro osteoclast cultures on dentine is an excellent system for the study of osteoclastogenesis and resorptive activity.^5^ Although M‐CSF and RANKL are now widely used to drive the cell maturation and differentiation required, the link between the bone mass of the source of precursor marrow cells and their osteoclastogenic potential remains only partially explored. Mouse models serve a vital role in the exploration of bone mass regulation; however, most studies use C57BL/6 mice with relatively low bone mass for the extraction of BM cells. In contrast, the STR/Ort mouse model of spontaneous OA is known to exhibit very high bone mass. Furthermore, the parental control‐CBA strain also has relatively high bone mass compared with other strains, yet is still significantly lower than STR/Ort mice.^12^ Weight does not differ much between these latter strains, yet the markedly high cortical and trabecular bone mass in STR/Ort mice is associated with elevated osteoblast numbers and impaired osteoclast function which is sufficient enough to lead a compressed BM space.^14^ In agreement, use of 8–10‐week‐old STR/Ort mice for marrow extraction proved extremely difficult, because of the narrowness of the medullary space compared with extractions from C57BL/6 and CBA animals of similar ages. This indicates that fewer BM cells can be derived from the STR/Ort mice with higher bone mass. Differences in BM cell yield were observed in 6‐ week‐old mice, where STR/Ort generate fourfold and twofold lower yields than C57BL/6 and CBA mice, respectively. This is consistent with the notion that bone mass inversely correlates with BM cell yield in these three mouse strains. Pasold et al.^14^ compared the BM cells in STR/Ort and C57BL/6 mice at 8 and 11 weeks of age to show extremely significant trends toward BM reduction in the former. Even though on gross pathologic examination STR/Ort mice otherwise show normal skeletal development, optical examination of dissected hind limbs suggests that there is already a lack of bone marrow at these early ages.^14^ This aligns with profound marrow compression linked to extramedullary hematopoiesis in STR/Ort mice.^14^ There is, however, a lack of studies comparing the relationship between compression of the BM cavity and bone mass across different strains, and therefore this relationship remains to be explored.

Our data also show that BM cells from STR/Ort mice produce far fewer mature multinucleated osteoclasts than from C57BL/6 and CBA mice; given identical starting precursor numbers and pro‐osteoclastogenic cytokine levels. This aligns with the findings of Pasold et al.^14^ in which BM from STR/Ort generated a lower number of TRAP+ osteoclasts compared to CB7BL/6. These data indicate that BM precursors from STR/Ort mice exhibit a defect in a late stage of osteoclastogenesis that is conserved during their isolation and in vitro maintenance, and is consistent with a deficit in *full* osteoclast differentiation potential or reduced sensitivity in vitro to M‐CSF and RANKL in these mice with highest bone mass. To determine whether this relative lack of differentiation in STR/Ort mouse BM precursor cells was due to differential M‐CSF and RANKL requirements, higher concentrations were used (5 ng/ml RANKL, 250 ng/ml M‐CSF). This confirmed that BM‐derived from STR/Ort mice could indeed differentiate to form mature osteoclasts but that numbers of TRAP+ cells were still lower than in C57BL/6 or CBA BM. This may be due to inherently lower expression levels for osteoclast differentiation‐linked genes such as c‐Fos, NFATc1, RANK or DC‐STAMP that have yet to be explored.

Pasold et al.^14^ also found fewer osteoclasts and resorption activity in BM cells derived from STR/Ort mice, in comparison to C57BL/6, leading them to speculate that the OA‐prone animals possessed a defect in final osteoclast differentiation. Our data suggest that this defective differentiation is potentially linked to high bone mass and reduced sensitivity to M‐CSF‐/RANKL in osteoclast precursors in this STR/Ort mouse strain. Furthermore, these data demonstrate that this lower osteoclastogenic potential is still somewhat *rescued* by increasing the concentration of M‐CSF and RANKL, to which osteoclast precursors are exposed. It is worth emphasising, that the data presented here uses an osteoclast system^5^ which differs somewhat from the earlier work by Pasold et al.^14^

The only study to our knowledge to report in vitro differences in mouse strains of diverging bone mass compared C3H/HeJ mice to C57BL/6 mice in which there was lower femoral bone density. These studies by Linkhart et al.,^6^ using coculture with Swiss/Webster mouse osteoblasts as a cell source of osteoclastogenic drive, found that BM cells from C57BL/6 produced more osteoclasts and resorbed more extensive pits on dentine in vitro than C3H/HeJ.^24^ These differences have been confirmed in vivo by another study comparing the same strains in which the higher bone mass C3H/HeJ mice exhibited fewer active (TRAP+) osteoclastic surfaces in the femur metaphysis compared with C57BL/6.^25^ These findings support the hypothesis that sensitivity to M‐CSF‐/RANKL‐induced in vitro osteoclastogenesis is indeed inferior in sources of BM‐derived from mice with progressively greater in vivo bone mass.

However, it is important to emphasise that the data reported here use an osteoclast system^5^ which differs somewhat from the earlier studies. Also, it is important to stress that the osteoclasts generated in these strains may simply undergo multinucleation in a manner related to their underpinning genetics and that by coincidence alone this is matched to their bone mass. Future studies might examine whether this holds true in other mouse strains. Inherent cellular sensitivity in the current osteoclast system to the serum, RANKL, M‐CSF reagents, and the dentine discs used may also impact these results, as it is known that these can exhibit batch variation and may even differ in their osteoclastogenic capacity dependent upon the supplier. Care was taken herein to utilise only a single source of all reagents in any comparison between the different mouse strains.

A secondary hypothesis tested was that this BM precursor sensitivity to exogenous factors extends concentration‐dependently to the inhibition of osteoclast formation and function by SFX‐01. Several studies had already explored the effects of SFN to conclude that it suppresses osteoclastogenesis.^19^, ^20^, ^21^, ^22^ Our work previously demonstrated that SFX‐01 also suppresses osteoclastogenesis in C57BL/6 mice. It should be emphasised however that apart from our studies, all those conducted previously used either RAW 264.7 cells (macrophage‐like transformed cell lines that mimic a restricted range of osteoclast behaviours) or cells derived only from C57BL/6 mice. The link between different mouse strains with their in vitro behaviour as well as their sensitivity to SFX‐01 has not been reported previously. Our data show for the first time that SFX‐01 exerts lower antiosteoclastogenic effects in BM cells from mice with higher bone mass. More specifically, 100 nM SFX‐01 was sufficient to exert a potent reduction in osteoclast number and resorption in cells from C57BL/6 mice, indicative of direct effects on in vitro maturation. This sensitivity to SFX‐01 was markedly lower in osteoclasts derived from CBA mice, which only showed similar levels of inhibition at higher 1 μΜ concentrations. Strikingly, STR/Ort‐derived BM cells were insensitive to SFX‐01 up to 1 μM and only showed a reduction in osteoclast formation and resorption at 2.5 μΜ. Whether this sensitivity extends to other regulators of osteoclast function, such as bisphosphonates, remains to be established but is an interesting area for future study. Together, our studies establish a lower sensitivity to M‐CSF‐/RANKL‐induced in vitro osteoclastogenic capacity in BM sources from the mice with higher bone mass. Furthermore, they show that this sensitivity extends to concentration‐dependent inhibitory effects of SFX‐01 on osteoclast formation and function. Interestingly, a study by Kyostio‐Moore et al.^26^ immunochemically demonstrated that specific regions of the periosteum label more strongly for osteoclast‐related markers, and that circulating levels of proinflammatory cytokines, including interleukin‐1 and interferon gamma, were higher in STR/Ort compared with CBA mice. These data are consistent with the notion that local and systemic oxidative and inflammatory marker status is linked to the OA that develops in this mouse strain. Together, with our data, this suggests that in vivo treatment of STR/Ort mice with compounds such as SFX‐01, with known antioxidant and anti‐inflammatory actions, may potentially limit these OA‐linked changes to reduce the OA that develops in these mice. To date, evidence supporting this potential for SFX‐01 treatment to alleviate the OA that develops in STR/Ort mice is, however, lacking as studies using SFX‐01 do not protect against OA progression but instead only serve to increase bone mass in STR/Ort mice.^11^

## CONFLICT OF INTEREST

The authors declare no conflict of interest.

## Data Availability

Data available on request from the authors.
